# Machine learning based on radiomics features combing B-mode transrectal ultrasound and contrast-enhanced ultrasound to improve peripheral zone prostate cancer detection

**DOI:** 10.1007/s00261-023-04050-5

**Published:** 2023-10-05

**Authors:** Ya Sun, Jingyang Fang, Yanping Shi, Huarong Li, Jiajun Wang, Jingxu Xu, Bao Zhang, Lei Liang

**Affiliations:** 1 Department of Ultrasound, Aerospace Center Hospital, 15 Yuquan Road, Haidian District, Beijing, China; 2 Department of Research Collaboration, R&D Center, Beijing Deepwise and League of PHD Technology Co., Ltd, Beijing, China; 3 Department of Urology, Aerospace Center Hospital, 15 Yuquan Road, Haidian District, Beijing, China

**Keywords:** Prostate cancer, Radiomics, Ultrasound, CEUS, Peripheral zone

## Abstract

**Purpose:**

To construct machine learning models based on radiomics features combing conventional transrectal ultrasound (B-mode) and contrast-enhanced ultrasound (CEUS) to improve prostate cancer (PCa) detection in peripheral zone (PZ).

**Methods:**

A prospective study of 166 men (72 benign, 94 malignant lesions) with targeted biopsy-confirmed pathology who underwent B-mode and CEUS examinations was performed. Risk factors, including age, serum total prostate-specific antigen (tPSA), free PSA (fPSA), f/t PSA, prostate volume and prostate-specific antigen density (PSAD), were collected. Time-intensity curves were obtained using SonoLiver software for all lesions in regions of interest. Four parameters were collected as risk factors: the maximum intensity (IMAX), rise time (RT), time to peak (TTP), and mean transit time (MTT). Radiomics features were extracted from the target lesions from B-mode and CEUS imaging. Multivariable logistic regression analysis was used to construct the model.

**Results:**

A total of 3306 features were extracted from seven categories. Finally, 32 features were screened out from radiomics models. Five models were developed to predict PCa: the B-mode radiomics model (B model), CEUS radiomics model (CEUS model), B-CEUS combined radiomics model (B-CEUS model), risk factors model, and risk factors-radiomics combined model (combined model). Age, PSAD, tPSA, and RT were significant independent predictors in discriminating benign and malignant PZ lesions (*P* < 0.05). The risk factors model combing these four predictors showed better discrimination in the validation cohort (area under the curve [AUC], 0.84) than the radiomics images (AUC, 0.79 on B model; AUC, 0.78 on CEUS model; AUC, 0.83 on B-CEUS model), and the combined model (AUC: 0.89) achieved the greatest predictive efficacy.

**Conclusion:**

The prediction model including B-mode and CEUS radiomics signatures and risk factors represents a promising diagnostic tool for PCa detection in PZ, which may contribute to clinical decision-making.

**Supplementary Information:**

The online version contains supplementary material available at 10.1007/s00261-023-04050-5.

## Introduction

Prostate cancer (PCa) is the most diagnosed urologic cancer and the second most frequently diagnosed malignant tumor in men worldwide [1]. In 2020, there were approximately 1,414,259 newly diagnosed cases of PCa worldwide, accounting for 375,304 cancer-related deaths. Although the incidence and mortality rates of PCa in China are relatively low, due to its large population, China accounts for 8.2% of the global new cases and 13.6% of the PCa-related deaths, highlighting the urgent need for increased attention to PCa [2, 3]. In 1980s, John McNeal described four distinct zones of the prostate: the peripheral zone (PZ), the central zone, the transition zone, and the anterior fibromuscular stroma, and 70–80% prostate carcinomas originate from the PZ [4, 5]. Diagnostic tools generally include digital rectal examination (DRE), prostate-specific antigen (PSA), multiparametric magnetic resonance imaging (mpMRI), transrectal ultrasound (TRUS), and prostate biopsy [6]. DRE is always incorporated as part of a routine primary care examination, and may result in a large number of false-positives leading to unnecessary invasive diagnostic methods [7]. PSA, including total PSA (tPSA) and free PSA (fPSA), is the most widely used procedure in clinical screening but has low specificity [8]. MpMRI has shown promising advances for patient selection and focal treatment guidance [9]. However, concerns remain regarding high costs, limited availability due to, for example, the presence of metal implants, and inconsistencies in the reliability of reporting despite the recognized Prostate Imaging Reporting and Data System (PI-RADS), which has its problems, namely, a slow learning curve and high operator disagreement [9, 10]. TRUS is cost-effective, practical, safe and widely available, and it especially plays an important role in the evaluation of the PZ [11, 12]. However, PCa can be either hypoechoic, isoechoic or hyperechoic on conventional B-mode TRUS, which is limited due to the heterogeneity of lesion echogenicity, resulting in limited value for PCa diagnosis, with a sensitivity and positive predictive value of approximately 11–35% and 27–57%, respectively [11–13]. Imaging-guided biopsy is still the standard diagnostic approach using a core needle mainly under ultrasound imaging guidance. However, the systematic biopsy is invasive, and more than 30% of patients experience side effects such as pain, infection, sepsis and bleeding [14]. Thus, we need to explore methods to increase PCa detection and to avoid unnecessary biopsy.

As a novel ultrasound technology, contrast-enhanced ultrasound (CEUS), which can reveal the dynamic patterns of blood flow in the cancer region, allows improved PCa visualization [15]. Specifically, through quantitative parameters of the measured time intensity curve (TIC), CEUS has produced encouraging results in previous studies [16, 17]. An improved PCa detection rate has been shown when applying CEUS for TRUS-guided biopsy compared to systematic biopsies without CEUS [18, 19], and a few methods involving tissue perfusion assessments have been proposed for PCa detection [20, 21].

Radiomics extracts several quantitative characteristics from various images, and has been demonstrated to have clinical value [22]. In previous studies, a growing number of studies have focused on MRI using radiomics or deep learning approaches [23]. Only a few studies have focused on ultrasound in PCa-related medical decisions [24–26], and no prospective studies have been made to evaluate the diagnostic performance of CEUS combined with parameters through a machine learning approach.

In this study, we aimed to construct machine learning models based on radiomic features combing conventional B-mode TRUS, CEUS, and risk factors to improve PZ PCa detection. To our knowledge, there has not been any similar prospective study that comprehensively predicts benign and malignant prostate lesions by using the aforementioned imaging and factors. In addition, we will develop a new nomogram prediction model for clinical use through a more convenient platform.

## Materials and methods

### Patients

A total of 176 inpatients from our hospital were prospectively included from January 2021 to August 2022. All of the patients were prospectively selected on the basis of suspicious PZ prostate lesions on TRUS. When a patient had multiple lesions, only the most suspicious one was evaluated. Finally, 166 PZ prostate lesions constituted the research group. The institutional ethics committee of aerospace central hospital approved this research.

**Fig. 1:**
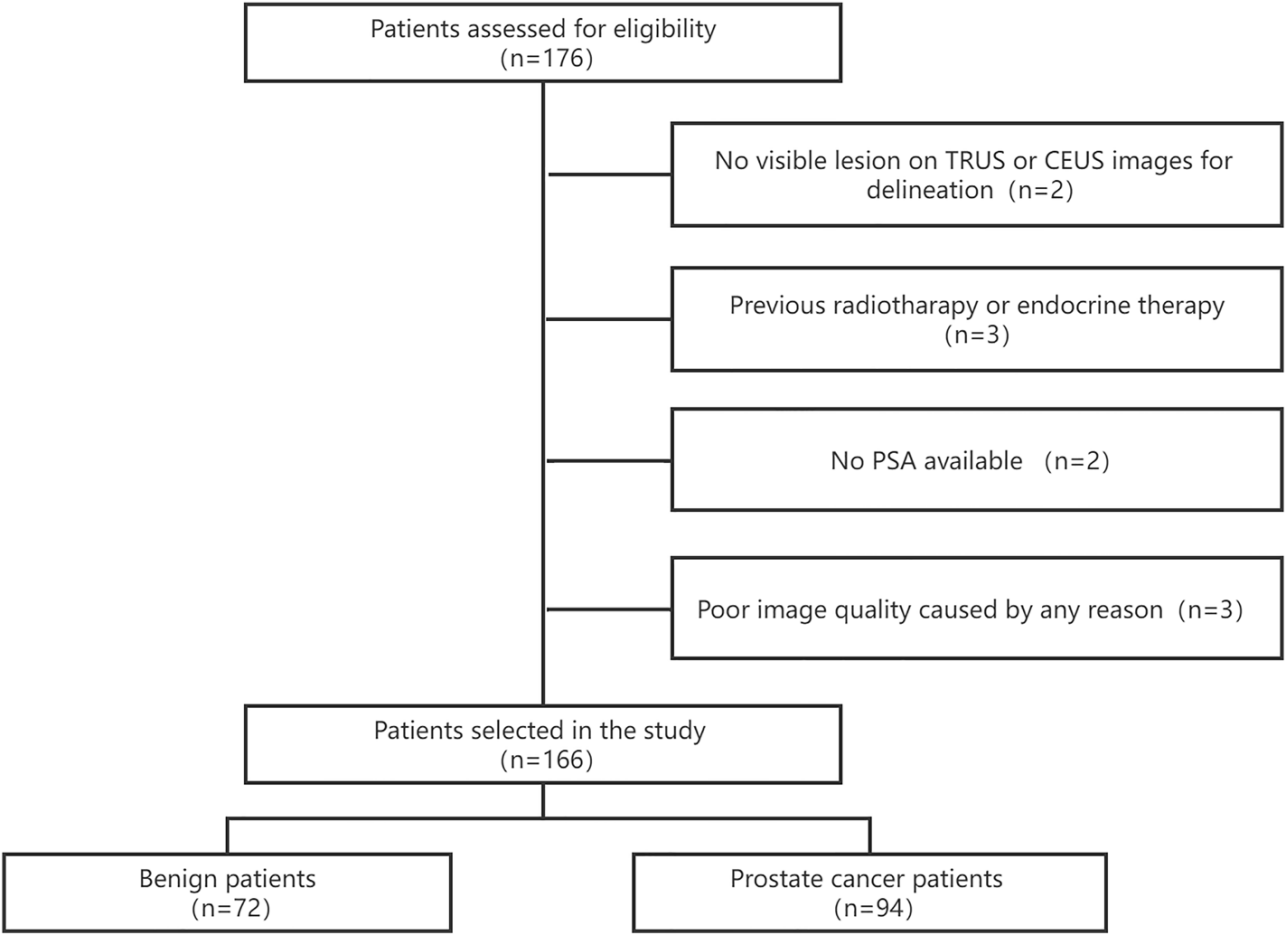
Diagram of patient inclusion for the study. *TRUS* transrectal ultrasound, *CEUS* contrast-enhanced ultrasound, *PSA* prostate-specific antigen

The inclusion criteria were as follows: (1) serum PSA testing (including tPSA, fPSA, and f/t PSA) within 1 month before biopsy; (2) B-mode and CEUS imaging showing a suspicious lesion in PZ; (3) clear B-mode and CEUS imaging, with the position of the lesion on the two different images showing good correspondence; and (4) available targeted biopsy results. The exclusion criteria were as follows: (1) incomplete imaging data for either B-mode or CEUS; (2) surgery, radiotherapy or endocrine therapy before the ultrasound examination; and (3) no satisfactory pathological results for the lesion biopsy. Fig. 1 shows a patient selection diagram.

Risk factors, including age, serum tPSA, fPSA, f/t PSA, prostate volume (PV) and prostate-specific antigen density (PSAD), DRE result (positive vs. negative) were acquired from the enrolled patients. PV was measured by TRUS, and PSAD was calculated as tPSA/PV. In addition, CEUS-related parameters were also included as risk factors, which are described in detail below.

### Ultrasound technique and biopsy

For the procedure, each patient underwent B-mode ultrasound, CEUS examination and biopsy in the left lateral decubitus position using an Aixplorer^®^ Ultrasound scanner (SuperSonic Imagine, Aix en Provence, France) equipped with an SE 12-3 transrectal probe. These procedures were performed by two sonographers (L.L. with 10 years of diagnostic prostate CEUS experience and 7 years of prostate biopsy experience and S.Y. with 5 and 3 years of such experience, respectively). All men or their legal guardians provided written informed consent.

**Fig. 2:**
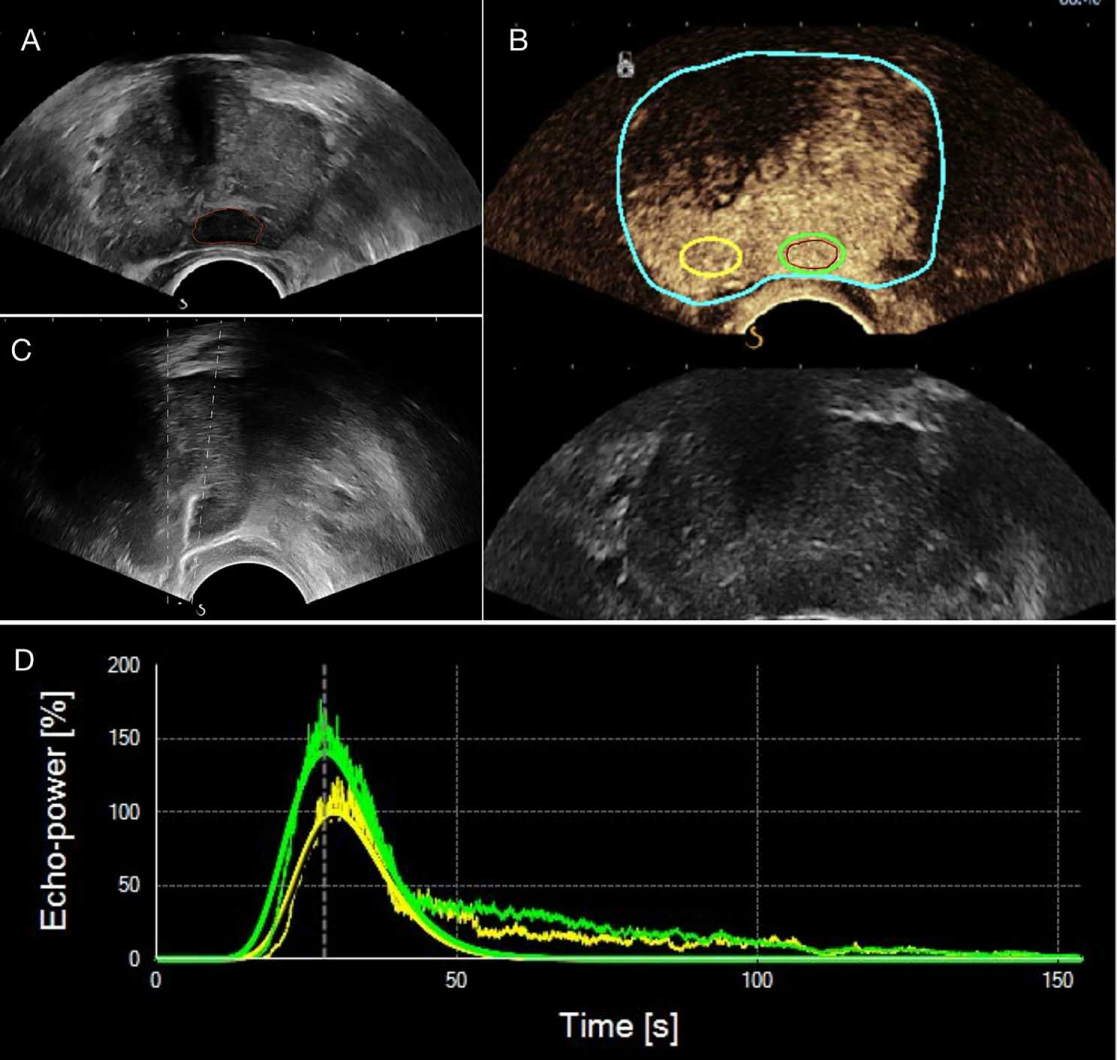
Example image of a 68-year-old PCa patient with an elevated PSA of 6.3 ng/mL and a hypoechoic lesion in the left PZ of the prostate. **a** B-mode, **b** CEUS mode, **c** target TRUS biopsy, and **d** the time-intensity curve. **a** and **b** represent ROI placement within the target lesion, which is depicted in the red circle. In **b**, the analysis and reference ROIs are encircled in green and yellow, respectively. *CEUS* contrast-enhanced ultrasound, *TRUS* transrectal ultrasound, *ROI* region of interest

B-mode US was first used to perform volumetry of the prostate gland and identify the best approach for the target lesion (Fig. 2a). For the CEUS examination, a low mechanical index of 0.04 were used. CEUS recording started after a standardized dose of 2.4 mL of the contrast agent SonoVue (Bracco, Milan, Italy) followed by a 5-mL saline flush was administered intravenously by bolus. Lesions were evaluated by continuous scanning for 120 s and DICOM video clips were stored. After all the ultrasound examinations, a BARD Biopsy gun (Tempe, Arizona, USA) with an 18-gauge biopsy needle and a penetration depth of 15 mm or 22 mm was applied to perform the biopsy procedure. After the systematic 12-core biopsies, a standardized biopsy scheme (base, mid or apex gland; left or right) and 1–2 needles were added to the suspicious lesion area (Fig. 2c). The specimens were placed into corresponding labeled bottles.

### CEUS evaluation

CEUS video clips were evaluated using TomTec’s SonoLiver software (1.0 version) to draw the TIC in this study (Fig. 2d). An ellipsoid region of interest (ROI) was placed within each target lesion. First, the ROI, including the lesion and the surrounding tissue, was delimited; second, for the ROI analysis, most of the lesion to be targeted compared to an area of the corresponding part of the contralateral side of the prostate parenchyma measuring approximately 1 cm was taken as a reference, which was located at the same depth as the reference ROI (Fig. 2b). TICs with a quality of fit > 75% were enrolled. The following SonoLiver output data were recorded: the maximum intensity (IMAX), the maximum echo-power with respect to that in the reference ROI; the rise time (RT), the wash-in time; the time to peak (TTP), which is defined as the instant at which the echo-power reaches the maximum; and the mean transit time (MTT) for the contrast agent in the ROI. These parameters were collected from clinical data, and the contrast image corresponding to the TTP was selected for inclusion in the next radiomics analysis.

### Segmentation and radiomics feature extraction

The images were imported into the open-source software ITK-SNAP (version 3.8.0), which was used to segment ROIs separately in the B-mode and CEUS images. The analyzed lesion was manually segmented by tracing the contour of the lesion on the B-mode image and the CEUS image at the time to peak by a radiologist. The red circle in Fig. 2b shows the lesion segmentation.

A total of 3306 features were extracted in this study from the Dr. Wise Multimodal Research Platform (https://keyan.deepwise.com) (Beijing Deepwise and League of PHD Technology Co., Ltd, 193 Beijing, China). The following standard classes of features were extracted: First-order statistics (19 features), shape-based features (10 features), gray-level co-occurrence matrix (GLCM) features (24 features), gray-level run length matrix (GLRLM) features (16 features), gray-level size zone matrix (GLSZM) features (16 features), neighboring gray tone difference matrix features (5 features) and gray-level dependence matrix features (14 features). These features are presented in the Appendix.

### Feature selection and model construction

Feature correlation analysis was used to implement feature selection. The B-mode radiomics model (B model), CEUS radiomics model (CEUS model), B-CEUS combined radiomics model (B-CEUS model), risk factors model, and risk factors-radiomics combined model (combined model) were built based on the features from each individual image and their combination. The fivefold cross validation method was used to verify the results of various models. In terms of selecting the most useful predictive combination of features, six kinds of feature-screening techniques (i.e., *F* test, the Pearson correlation coefficient, mutual information, the L1-based model, tree-based models, and recursive feature elimination) were adopted. Each method was selected for feature screening to build the model one by one. Finally, the L1-based model was demonstrated to be the most effective method for discriminating benign and malignant lesions. A logistic regression model was used to build each radiomics signature, and then a formula called radiomics score (Rad-score) was generated by analyzing the regression characteristics weighted by their coefficients. To build the risk factors model, univariate and multivariate logistic regression analyses incorporating the risk factors (i.e., age, PV, PSAD, tPSA, fPSA, f/t PSA, RT, TTP, MTT, and Imax) were used. An integrated combined model was then built.

### Statistical analysis

Quantitative data are presented as the mean ± standard deviation. An independent *t* test or the Mann–Whitney *U* test was implemented to analyze the continuous variables, including nonnormally distributed data. When building the risk factors model, univariate logistic regression was applied first to choose the independent predictors with *P* < 0.05, and then multivariable logistic regression analysis was adopted to identify these factors in the combination of features. The enter stepwise selection method was applied in this step. The odds ratio (OR) was used to indicate the degree of risk. The diagnostic efficiency of a predictor was evaluated by its sensitivity, specificity, and accuracy. Receiver operating curves (ROC) and the area under the curves (AUCs) with 95% confidence intervals (95% CIs) of the different models were obtained to assess their diagnostic performance and were compared using the Delong test. The nomogram of the combined model and decision curve analysis (DCA) was established to facilitate clinical decision-making. Statistical analyses were performed using R software (version 4.0.2) and SPSS (version 23.0). *P *< 0.05 in two-tailed analyses was used to define statistical significance.

## Results

In total, 166 patients (mean age, 73.9 ± 8.8 years; age range, 47–83 years) were enrolled. The median diameter of the lesions was 7.8 mm (min–max 4.6–35.5 mm). The Gleason scores (GSs) of all patients were as follows: 3 + 3 = 6 (30 cases); 3 + 4 = 7 (24 cases); 4 + 3 = 7 (45 cases); and > 4 + 3 (67 cases). Based on the univariate logistic regression analysis, significant differences in age, PV, PSAD, tPSA, fPSA, and f/t PSA and in the presence of the CEUS parameter RT were identified between the benign and malignant groups (*P* < 0.05). No differences in the distribution of the remaining three parameters of CEUS (TTP, MTT, and Imax) were noted between the two groups (*P* > 0.05). In the multivariate logistic analysis, age, PSAD, tPSA, and RT were significant independent predictors (*P* < 0.05). Patient demographic and clinical characteristics are shown in Table 1.

**Table 1:** Characteristics of patients in the benign and malignant groups.

	Benign group (N = 72)	Malignant group (N = 94)	Univariate logistic analysis results		Multivariate logistic analysis results	
			OR (95% CI)	*P* value	OR (95% CI)	*P* value
Age (years)	71.9 ± 9.0	76.0 ± 8.3	1.058 (1.009, 1.109)	0.020	1.078 (1.009, 1.152)	0.026
PV (mL)	63.9 ± 36.4	50.0 ± 28.4	0.985 (0.971, 0.999)	0.034	1.020 (0.994, 1.047)	0.139
PSAD (ng/mL^2^)	0.2 ± 0.2	1.0 ± 1.4	45.630 (3.462, 601.372)	0.004	35,162.63 (11.110, 11,287,346)	0.011
tPSA (ng/mL)	11.0 ± 12.5	55.2 ± 137.9	1.041 (1.011, 1.071)	0.007	0.930 (0.875, 0.989)	0.020
fPSA (ng/mL)	2.3 ± 4.1	6.6 ± 9.7	1.104 (1.018, 1.196)	0.017	0.945 (0.743, 1.203)	0.647
f/t PSA (%)	18.8 ± 7.9	14.3 ± 7.9	0.927 (0.878, 0.978)	0.005	0.910 (0.808, 1.024)	0.117
RT (s)	15.0 ± 4.7	11.6 ± 3.2	0.796 (0.706, 0.898)	0.000	0.781 (0.644, 0.947)	0.012
TTP (s)	35.15 ± 6.9	32.85 ± 8.9	0.961 (0.910, 1.015)	0.151		
MTT (s)	38.53 ± 10.6	32.8 ± 7.0	0.921 (0.880, 0.975)	0.004	0.977 (0.898, 1.062)	0.582
IMAX (%)	125.3 ± 82.0	165.7 ± 112.4	1.005 (1.000, 1.009)	0.056		
Positive DRE	41	54	3.232 (1.077, 9.703)	0.134		

*PV* prostate volume, *PSAD* prostate-specific antigen density, *PSA* prostate-specific antigen, *tPSA* total PSA, *fPSA* free PSA, *IMAX* maximum intensity, *RT* rise time, *TTP* time to peak, *MTT* mean transit time, *DRE* digital rectal examination

**Fig. 3:**
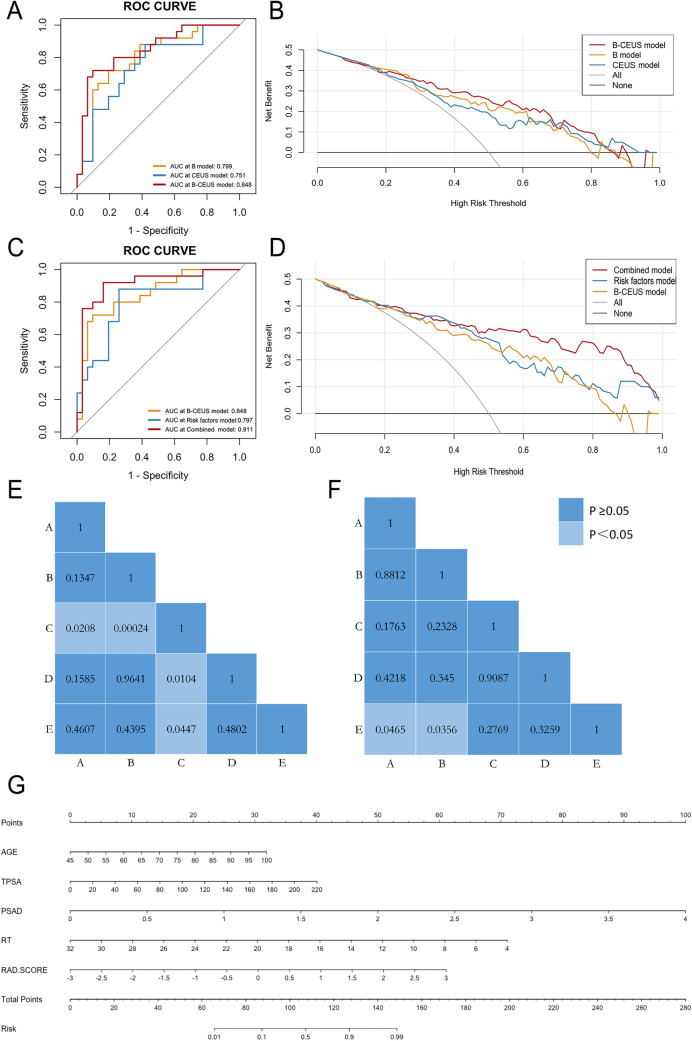
The performance of the different models for PZ PCa prediction. **a** ROC curves and **b** DCA curves of the B model, CEUS model, and B-CEUS model in the validation cohort. **c** ROC curves and **d** DCA curves of the risk factors model, B-CEUS model, and combined model in the validation cohort. **e**, **f** Delong test for the AUCs of different models. a = B model; b = CEUS model; c = B-CEUS model; d = risk factors model; e = combined model. **e** Training cohort; **f** Validation cohort. **g** Nomogram of the combined model for PZ PCa prediction. *ROC* receiver operating curves, *DCA* decision curve analysis, *PCa* prostate cancer, *AUC* area under the curve, *CEUS* contrast-enhanced ultrasound

1653 B-mode features were reduced to 20 risk predictors in the training set, and the B-mode Rad-score was obtained. The same steps were also completed for the CEUS training set, and 11 risk predictors together with the CEUS Rad-score was obtained. Then, the 3306 features of the two modes were reduced to 32 related features, including 20 features related to B-mode and 12 to CEUS, and the multiparametric B-CEUS Rad-score was obtained, as presented in the Appendix. ROC curves were used to compare different models’ ability to discriminate between benign and malignant lesions. In the training set, the AUC values of B-mode radiomics, CEUS radiomics, and B-CEUS combined radiomics were 0.94 (CI 0.89–0.98), 0.87 (CI 0.81–0.94), and 0.97 (CI 0.95–1.0), respectively. In the validation set, the AUC values of the machine learning models were 0.79 (CI 0.71–0.88), 0.78 (CI 0.70–0.87), and 0.83 (CI 0.76–0.91), respectively, as shown in Fig. 3a.

For PCa prediction, the sensitivity, specificity, accuracy and AUC of risk factors model were 0.76, 0.81, 0.78, and 0.87, respectively in the training set. In the validation set, they were 0.69, 0.77, 0.74, and 0.84, respectively. The risk factors-radiomics combined model displayed a good predictive capacity with an AUC of 0.91 in the training group and the best predictive capacity with an AUC of 0.89 in the validation group, as shown in Fig. 3c. The specific indicators of diagnostic efficacy for these models are shown in Table 2.

**Table 2:** The diagnostic performance of the various models for predicting prostate cancer.

	Sensitivity	Specificity	Accuracy	AUC (95% CI)
B-mode radiomics model				
Training set	0.88	0.91	0.89	0.94 (0.89–0.98)
Validation set	0.67	0.78	0.73	0.79 (0.71–0.88)
CEUS radiomics model				
Training set	0.82	0.69	0.75	0.87 (0.81–0.94)
Validation set	0.76	0.65	0.70	0.78 (0.70–0.87)
B-CEUS combined radiomics model				
Training set	0.92	0.89	0.90	0.97 (0.95–1.0)
Validation set	0.82	0.74	0.78	0.83 (0.76–0.91)
Risk factors model				
Training set	0.76	0.81	0.78	0.87 (0.81–0.94)
Validation set	0.69	0.77	0.74	0.84 (0.76–0.92)
Risk factors-radiomics combined model				
Training set	0.80	0.87	0.84	0.91 (0.85–0.97)
Validation set	0.80	0.83	0.82	0.89 (0.83–0.96)

*CEUS* contrast-enhanced ultrasound, *AUC* area under the curve

The Delong test (Fig. 3e, f) revealed that the B-CEUS model performed the best in the training set, and in the validation set, significant differences were observed between the AUCs of the combined model and the single-imaging morality radiomics model (B model, *P* < 0.05; CEUS model, *P* < 0.05). The combined model also showed not only the highest specificity and accuracy (0.83 and 0.82, respectively) but also high sensitivity (0.80) for PCa prediction.

According to the DCA (Fig. 3b, d), using the combined model added more benefit if the high-risk threshold probability was > 40% within a wide range. A nomogram including age, tPSA, PSAD, RT and Rad-score was built (Fig. 3g). The AUCs of this diagnostic nomogram were 0.91 (0.85–0.97) in the training set and 0.89 (0.83–0.96) in the validation set. The accuracies were 0.84 in the training cohort and 0.82 in the testing cohort.

## Discussion

Our study presents new opportunities for PZ PCa detection. A combined model was finally set up with the goal of establishing an easy-to-use tool.

Previous studies have presented controversial results regarding hypoechoic lesions in the detection of PZ PCa. Kwang Suk Lee et al. [27] reported a high proportion (76.9%) of high-grade GSs for hypoechoic PCa lesions, and hypoechoic lesions generally have worse pathologic differentiation with increasing size. Nakano Junqueira et al. [28] showed that patients with hypoechoic lesions who underwent prostatectomy had significantly worse outcomes than those who did not. A hypoechoic lesion was defined as a region with a lower grayscale value than the surrounding tissue [29]. In our study, the suspicious lesions mostly included hypoechoic lesions and few isoechoic or mixed lesions. However, hypoechoic lesions may become hyperechoic, isoechoic, or mixed lesions depending on whether they grow, invade other issues, or develop calcification [11, 15].

Li et al. [19] noted the indicators predicting suspicious lesions when using CEUS: (1) hypoechoic lesions in the peripheral zone showed high enhancement, (2) the peak intensity of enhancement within the lesion was increased, (3) asymmetric enhancement, etc. Various enhancement patterns can coexist due to the uniformity of microvessel density. Notably, we chose the CEUS image of the TTP moment to segment ROIs because the image at this moment reflected the section with the most abundant blood supply due to its microvessel density (MVD). Bono et al. [30] detected a significant difference in the MVD of PCa among different groups of GS scores, and a higher GS score corresponded to higher MVD in PCa. Additionally, in Andreas Maxeiner’s study [17], within a subgroup analysis [> vs. ≤ 3 + 4 = 7a (ISUP 2)], peak enhancement (PE) [a.u] showed statistical significance with the software used (VueBox, Bracco). As a wash-in parameter index, PE exactly reflected the real maximum echo-power of the target lesion at the TTP moment. However, in our software TomTec’s SonoLiver, only one related index, ‘Imax’, was identified, which is a percentage, reflecting the ratio of the peak enhancement of the target ROI and the reference ROI at the peak time, rather than the echo-power itself. Therefore, the CEUS image of the TTP moment was selected for the radiomic analysis.

Jiang et al. [18] showed that the peak intensity of PCa was significantly higher than that of benign prostatic hyperplasia (BPH) lesions. BPH is the most common benign lesion in the prostate and corresponds to a histopathological hyperplastic process causing glandular-epithelial growth and stromal/muscle tissue in the prostate, especially in the periurethral region of the prostate [27]. Nevertheless, the pathological changes associated with PCa mostly originate from the growth of cancer cells and changes in the extracellular space [8]. Moreover, the low specificities may also be explained by prostatitis, which causes high enhancement on CEUS [19].

Radiomics can reflect the distribution of various cell components, fluid, collagen, and fibromuscular matrix in different prostate lesions, which can provide value through quantitative analysis of different imaging features. According to the high weights of the characteristic coefficients, “Cluster Shade” and “Zone Entropy” on CEUS imaging and “Zone Entropy” and “Dependence Non Uniformity Normalized (DN)” on conventional ultrasound imaging were relatively vital characteristics for PCa identification in our study. “Cluster shade” is a measure of the skewness and uniformity of the GLCM. A higher cluster shade implies greater asymmetry about the mean; it was higher in PCa than in benign tumors. ZoneEntropy measures the uncertainty/randomness in the distribution of zone sizes and gray levels. A higher value on either CEUS or B-mode US indicates greater heterogeneity in the texture patterns, which can be used as a predictor of PCa. DN measures the similarity of dependence throughout an image, with a lower value indicating more homogeneity among dependencies in the image.

As for risk factors, those with statistical significance are consistent with those obtained by performing univariate analysis followed by multivariate analysis. Given the considerable lack of evidence supporting its efficacy, although the DRE is commonly performed to screen for prostate cancer, researches [7] recommend against routine performance of DRE to screen for prostate cancer in the primary care setting. Previous studies have demonstrated that age and PSA levels are related to prostate cancer. Junxiao Liu et al. [31] reported that tPSA (AUC = 0.74), fPSA (AUC = 0.68), PV (AUC = 0.62), and PSAD (AUC = 0.77) were significant predictors in the detection and localization of prostate cancer from suspicious mpMRI results, and PSAD and tPSA had higher diagnostic accuracy than other single parameters, which is consistent with our study. Our research indicated that PV, PSAD, tPSA, fPSA and f/t PSA were all significant factors by univariate logistic analysis, and age, PSAD, and tPSA were independent risk factors by multivariable logistic regression analysis.

Except for these usual clinical risk factors, we also incorporated CEUS parameters in the analysis. A common problem with CEUS is the examiner’s subjective judgment. For example, early enhancement and the peak intensity determination relied on the subjective judgment of the radiologist. Therefore, quantitative measurements are needed. According to Jung’s study [16], tumor detection was possible in 85.3% and 73.5% of cases by evaluating RT and MTT, respectively. Baur’s research revealed that the TTP showed significant differences between benign lesions and PCa (AUC 0.65) [32]. In our study, RT and MTT demonstrated significant performance, as reported in the literature, which reflected the hypothesized hypervascularity owing to angiogenesis during tumor growth [30], and RT was the only independent factor by multivariable logistic regression analysis. The TTP showed no significance in our analysis, which may be due to sample differences.

Radiomic models have mostly been used with mpMRI to discriminate PCa, predict the GS score, identify lesions, and plan radiotherapy. However, studies on ultrasound-based radiomics are rare. Lorusso’s et al. retrospectively analyzed data from 64 patients with PCa followed by a computerized artificial neural network analysis of the TRUS based on an artificial intelligence, and on a per-sectors analysis, the sensitivity, specificity and accuracy were 62%, 81%, and 78% respectively [26]. Wildeboer et al. studied 48 patients demonstrating that multiparametric machine learning combined with B-mode, shear-wave elastography (SWE), and CEUS radiomics achieved ROC curves of 0.75 and 0.90 for PCa and significant PCa, respectively [24]. In our study, a more widely used pyradiomic approach was adopted together with risk factors, which also demonstrated that the Rad-score can improve diagnostic performance and the clinical net benefit in PCa distinction.

To date, nomograms have been widely used in the medical field. In our research, by using risk scores, we validated a combined risk factors-radiomics combined nomogram including age, tPSA, PSAD, RT, and Rad-score to diagnose PCa, providing a more quantifiable, distinct, and individualized auxiliary tool to clinicians.

Despite a positive role for the prediction model in PZ PCa detection, we acknowledge further limitations of the present study. First, this is a single-center analysis with a small population, and although the radiomics features differed between different GSs [33], they were not separated in our research because of the sample size. Therefore, larger, multicenter datasets are needed. In addition, manual segmentation might influence stability and repeatability, and automatic segmentation may be used to solve this problem in the future. Furthermore, ultrasound may be restricted by a large prostate volume because of the far-field attenuation effect. Last, the quantitative perfusion analysis relied on one cross-sectional image, providing limited information, which can possibly be overcome by 3D/4D-ultrasound probes.

## Conclusion

In conclusion, we developed radiomics models to discriminate PZ benign and malignant lesions. The nomogram incorporating both the radiomic signature and clinical risk characteristics will better contribute to accurate identification of PZ PCa lesions with intuitive evaluation indicators. Further studies with large sample sizes from multiple centers are necessary to validate our primary results.

### Supplementary Information

Below is the link to the electronic supplementary material.

Supplementary file1 (DOCX 16 KB)

## Data Availability

The original contributions presented in the study are included in the article. Further inquiries can be directed to the corresponding authors.
