# Supplementary material for: Machine learning based on radiomics features combing B-mode transrectal ultrasound and contrast-enhanced ultrasound to improve peripheral zone prostate cancer detection
Source: Abdom Radiol (NY). 2023 Oct 5;49(1):141–50. doi: 10.1007/s00261-023-04050-5 (PMC10789837; doi:10.1007/s00261-023-04050-5)
Supplement: Supplementary file 1 — Supplementary file1 (DOCX 16 KB) [file 261_2023_4050_MOESM1_ESM.docx]

Appendix 1.

Table 1. Radiomic Features

| Feature classes | N | Feature definitions |
| --- | --- | --- |
| First-order statistics features | 19 | Energy; Total Energy; Entropy; Minimum; 10th percentile; 90th percentile; Maximum; Mean; Median; Interquartile Range; Range; Mean Absolute Deviation (MAD); Robust Mean Absolute Deviation (rMAD); Root Mean Squared (RMS); Standard Deviation; Skewness; Kurtosis; Variance; Uniformity |
| Shape-based (2D) features | 10 | Mesh Surface; Pixel Surface; Perimeter; Perimeter to Surface ratio; Sphericity; Spherical Disproportion; Maximum 2D diameter; Major Axis Length; Minor Axis Length; Elongation |
| Gray-level co-occurrence matrix (GLCM) features | 24 | Autocorrelation; Joint Average; Cluster Prominence; Cluster Shade; Cluster Tendency; Contrast; Correlation; Difference Average; Difference Entropy; Difference Variance; Joint Energy; Joint Entropy; Informational Measure of Correlation (IMC) 1; Informational Measure of Correlation (IMC) 2; Inverse Difference Moment (IDM); Maximal Correlation Coefficient (MCC); Inverse Difference Moment Normalized (IDMN); Inverse Difference (ID); Inverse Difference Normalized (IDN); Inverse Variance; Maximum Probability; Sum Average; Sum Entropy; Sum of Squares |
| Gray-level run length matrix (GLRLM) features | 16 | Short Run Emphasis (SRE); Long Run Emphasis (LRE); Gray Level Non-Uniformity (GLN); Gray Level Non-Uniformity Normalized (GLNN); Run Length Non-Uniformity (RLN); Run Length Non-Uniformity Normalized (RLNN); Run Percentage (RP); Gray Level Variance (GLV); Run Variance (RV); Run Entropy (RE); Low Gray Level Run Emphasis (LGLRE); High Gray Level Run Emphasis (HGLRE); Short Run Low Gray Level Emphasis (SRLGLE); Short Run High Gray Level Emphasis (SRHGLE); Long Run Low Gray Level Emphasis (LRLGLE); Long Run High Gray Level Emphasis (LRHGLE) |
| Gray-level size zone matrix (GLSZM) features | 16 | Small Area Emphasis (SAE); Large Area Emphasis (LAE); Gray Level Non-Uniformity (GLN); Gray Level Non-Uniformity Normalized (GLNN); Size-Zone Non-Uniformity (SZN); Size-Zone Non-Uniformity Normalized (SZNN); Zone Percentage (ZP); Gray Level Variance (GLV); Zone Variance (ZV); Zone Entropy (ZE); Low Gray Level Zone Emphasis (LGLZE); High Gray Level Zone Emphasis (HGLZE); Small Area Low Gray Level Emphasis (SALGLE); Small Area High Gray Level Emphasis (SAHGLE); Large Area Low Gray Level Emphasis (LALGLE); Large Area High Gray Level Emphasis (LAHGLE) |
| Gray-level dependence matrix features | 14 | Small Dependence Emphasis (SDE); Large Dependence Emphasis (LDE); Gray Level Non-Uniformity (GLN); Dependence Non-Uniformity (DN); Dependence Non-Uniformity Normalized (DNN); Gray Level Variance (GLV); Dependence Variance (DV); Dependence Entropy (DE); Low Gray Level Emphasis (LGLE); High Gray Level Emphasis (HGLE); Small Dependence Low Gray Level Emphasis (SDLGLE); Small Dependence High Gray Level Emphasis (SDHGLE); Large Dependence Low Gray Level Emphasis (LDLGLE); Large Dependence High Gray Level Emphasis (LDHGLE) |
| Neighboring gray tone difference matrix features | 5 | Coarseness; Contrast; Busyness; Complexity; Strength |

Appendix 2. Formulas of three radiomics models

B-mode Rad-score:

-0.6090 x wavelet-LHH_glrlm_ShortRunHighGrayLevelEmphasis@b + 0.3164 x square_glrlm_RunVariance@b + -0.4106 x gradient_glszm_SizeZoneNonUniformityNormalized@b + 0.2383 x wavelet-HHL_gldm_LargeDependenceEmphasis@b + 0.5495 x wavelet-HHH_glszm_GrayLevelNonUniformityNormalized@b + 0.4525 x wavelet-LHH_glszm_GrayLevelNonUniformityNormalized@b + 0.6475 x wavelet-HHL_glszm_ZoneEntropy@b + 0.5554 x wavelet-LHH_gldm_DependenceNonUniformityNormalized@b + -0.1801 x original_shape_Elongation@b + 0.2485 x squareroot_gldm_DependenceNonUniformityNormalized@b + -0.1291 x wavelet-HHH_glszm_GrayLevelNonUniformity@b + 0.0783 x wavelet-HLL_firstorder_Range@b + 0.0522 x exponential_gldm_LargeDependenceHighGrayLevelEmphasis@b + 0.3249 x wavelet-HHH_glrlm_GrayLevelVariance@b + -0.4215 x wavelet-LHH_glszm_LowGrayLevelZoneEmphasis@b + -0.2937 x wavelet-LHH_firstorder_Skewness@b + -0.2670 x original_gldm_DependenceNonUniformityNormalized@b + -0.1023 x wavelet-LHH_glszm_SizeZoneNonUniformityNormalized@b + -0.2138 x wavelet-LHH_glcm_SumSquares@b + -0.3116 x wavelet-HHL_ngtdm_Complexity@b + -0.1132

CEUS Rad-score:

0.3518 x wavelet-HHH_firstorder_Kurtosis@ceus + 0.6484 x wavelet-HHH_glszm_ZoneEntropy@ceus + -0.3898 x wavelet-LHH_gldm_SmallDependenceLowGrayLevelEmphasis@ceus + 0.4835 x exponential_glcm_ClusterShade@ceus + 0.2762 x wavelet-LHL_glcm_InverseVariance@ceus + -0.2544 x original_glcm_ClusterShade@ceus + 0.0963 x wavelet-LLH_glcm_Idmn@ceus + -0.4484 x square_glszm_SmallAreaEmphasis@ceus + 0.0015 x square_ngtdm_Strength@ceus + -0.3637 x wavelet-LHH_glcm_Imc2@ceus + 0.2089 x wavelet-LHH_glszm_GrayLevelNonUniformityNormalized@ceus + 0.0000

Multiparametric B-CEUS Rad-score:

-0.2687 x wavelet-LHH_glrlm_ShortRunHighGrayLevelEmphasis@b + 0.1693 x square_glrlm_RunVariance@b + 0.1923 x wavelet-HHH_firstorder_Kurtosis@ceus + 0.2497 x wavelet-HHH_glszm_ZoneEntropy@ceus + -0.1384 x wavelet-LHH_gldm_SmallDependenceLowGrayLevelEmphasis@ceus + -0.2250 x gradient_glszm_SizeZoneNonUniformityNormalized@b + 0.3002 x exponential_glcm_ClusterShade@ceus + 0.0655 x wavelet-HHL_gldm_LargeDependenceEmphasis@b + 0.1305 x wavelet-LHL_glcm_InverseVariance@ceus + 0.1341 x wavelet-HHH_glszm_GrayLevelNonUniformityNormalized@b + 0.1423 x wavelet-LHH_glszm_GrayLevelNonUniformityNormalized@b + 0.2101 x wavelet-HHL_glszm_ZoneEntropy@b + 0.2069 x wavelet-LHH_gldm_DependenceNonUniformityNormalized@b + -0.1021 x original_glcm_ClusterShade@ceus + 0.1633 x wavelet-LLH_glcm_Idmn@ceus + -0.1718 x original_shape_Elongation@b + 0.1621 x squareroot_gldm_DependenceNonUniformityNormalized@b + -0.1845 x square_glszm_SmallAreaEmphasis@ceus + -0.0266 x square_ngtdm_Strength@ceus + 0.0208 x wavelet-HHH_glszm_GrayLevelNonUniformity@b + 0.0430 x wavelet-HLL_firstorder_Range@b + -0.0989 x wavelet-LHH_glcm_Imc2@ceus + 0.0633 x exponential_gldm_LargeDependenceHighGrayLevelEmphasis@b + 0.1447 x wavelet-LHH_glszm_GrayLevelNonUniformityNormalized@ceus + 0.0954 x wavelet-HHH_glrlm_GrayLevelVariance@b + -0.0418 x wavelet-LHH_glszm_LowGrayLevelZoneEmphasis@b + -0.1626 x wavelet-LHH_firstorder_Skewness@b + -0.1587 x original_gldm_DependenceNonUniformityNormalized@b + -0.0311 x wavelet-LHH_glszm_SizeZoneNonUniformityNormalized@b + -0.1775 x wavelet-LHH_glcm_SumSquares@b + -0.0264 x wavelet-LHH_firstorder_Kurtosis@b + -0.1549 x wavelet-HHL_ngtdm_Complexity@b + 0.0000
